# The current use of glaucoma virtual clinics in Europe

**DOI:** 10.1038/s41433-022-02111-5

**Published:** 2022-06-11

**Authors:** Matthew Azzopardi, Verena Prokosch-Willing, Manuele Michelessi, Antonio Maria Fea, Francesco Oddone, Karl Mercieca

**Affiliations:** 1 Birmingham and Midland Eye Centre, Dudley Road, Birmingham, UK; 2 Department of Ophthalmology, University of Cologne, Kerpener Str. 62, 50937 Köln, Germany; 3 IRCCS Fondazione Bietti, Rome, Italy; 4 Dipartimento di Scienze Chirurgiche, Universita degli Studi di Torino, Torino, Italy; 5 University Hospital Bonn, Eye Clinic, Ernst Abbe Strasse 2, Bonn, Germany; 6 Faculty of Biology, Medicine and Health, University of Manchester, Manchester, UK

**Keywords:** Health services, Glaucoma

## Abstract

**Objectives:**

To assess and describe current utilisation, characteristics and perspectives on virtual glaucoma clinics (VGCs) amongst European glaucoma specialists.

**Methods:**

Cross-sectional, anonymized, online questionnaire distributed to all European Glaucoma Society-registered specialists. Questions were stratified into five domains: Demographics, Questions about VGC use, Questions for non-VGC users, COVID-19 effects, and VGC advantages/disadvantages.

**Results:**

30% of 169 participants currently use VGCs, with 53% based in the United Kingdom. Of those using VGCs, 85% reported higher patient acceptance compared to traditional care. The commonest virtual model was asynchronous remote monitoring (54%). Nurses (49%) and ophthalmic technicians (46%) were mostly responsible for data collection, with two-thirds using a mixture of professionals. Consultant ophthalmologists were the main decision-makers in 51% of VGCs. Preferred cohorts were: ocular hypertension (85%), glaucoma suspects (80%), early/moderate glaucoma in worse eye (68%), stable glaucoma irrespective of treatment (59%) and stable glaucoma on monotherapy (51%). Commonest investigations were: IOP (90%), BCVA (88%), visual field testing (85%) and OCT (78%), with 33 different combinations. Reasons for face-to-face referral included: visual field progression (80%), ‘above-target’ IOP (63%), and OCT progression (51%). Reasons for not using VGCs included: lack of experience (47%), adequate systems in place (42%), no appropriate staff (34%) and insufficient time/money (34%). 55% of non-VGC users are interested in their use with 38% currently considering future implementation. 83% stated VGC consultations have increased during the COVID-19 pandemic; 86% of all participants felt that the pandemic has highlighted the importance of VGCs.

**Conclusions:**

A significant proportion of European glaucoma units are currently using VGCs, while others are considering implementation. Financial reimbursement and consensus guidelines are potentially crucial steps in VGC uptake.

## Introduction

The management of chronic diseases requiring lifelong follow-up is an important and pressing issue for health services. Glaucoma is the leading cause worldwide of irreversible blindness [1], and is estimated to afflict 111.8 million people worldwide by 2040 [2]. With increasing age, the burden rises. In 2018, 32.8% of the total EU-28 population was aged over 55 years, with a projected increase to 40.6% by 2050 [3]. All of the above, along with the use of newer diagnostic techniques in primary care [4], have served to increase the glaucoma-related burden.

Over the past two decades, teleophthalmology and ‘virtual clinics’ in glaucoma care have evolved, with the advent of SARS-CoV-2 presenting new challenges. Ophthalmology outpatient visits have been dramatically curtailed with a rebound increase expected as the pandemic improves [5]. Telemedicine could therefore play a vital role in the continued delivery of ophthalmology care [6].

The aim of this European-wide study was to quantify, describe and evaluate the use of virtual glaucoma clinics (VGCs) across Europe. In particular, we sought to compare how and for whom these clinics are used, and to evaluate the perceived results. We also aimed to compare differences in organisational structure. Where VGCs are not used, we delved into the possible reasons, overall perceptions and potential barriers. Finally, we also assessed whether the COVID-19 pandemic changed clinicians’ perception of VGCs.

## Methods

A cross-sectional anonymous online survey (SurveyMonkey^®^) was distributed to all glaucoma specialists registered with the European Glaucoma Society (EGS). The survey remained open for a total period of 2 weeks (16th to 30th April 2021), with two further email reminders. Participants were asked specific questions about use of VGCs in their hospital, stratified into five main domains (respondents’ demographics, questions for those currently running VGCs, questions for those not currently running VGCs, effect of COVID-19 on the need for VGCs, disadvantages and advantages of VGCs), each with related stem questions. Participants had to be based in European countries. For the purpose of this study, Turkey and Russia were considered to be part of the European sphere. Data obtained was analysed using SPSS software.

## Results

**Fig. 1:**
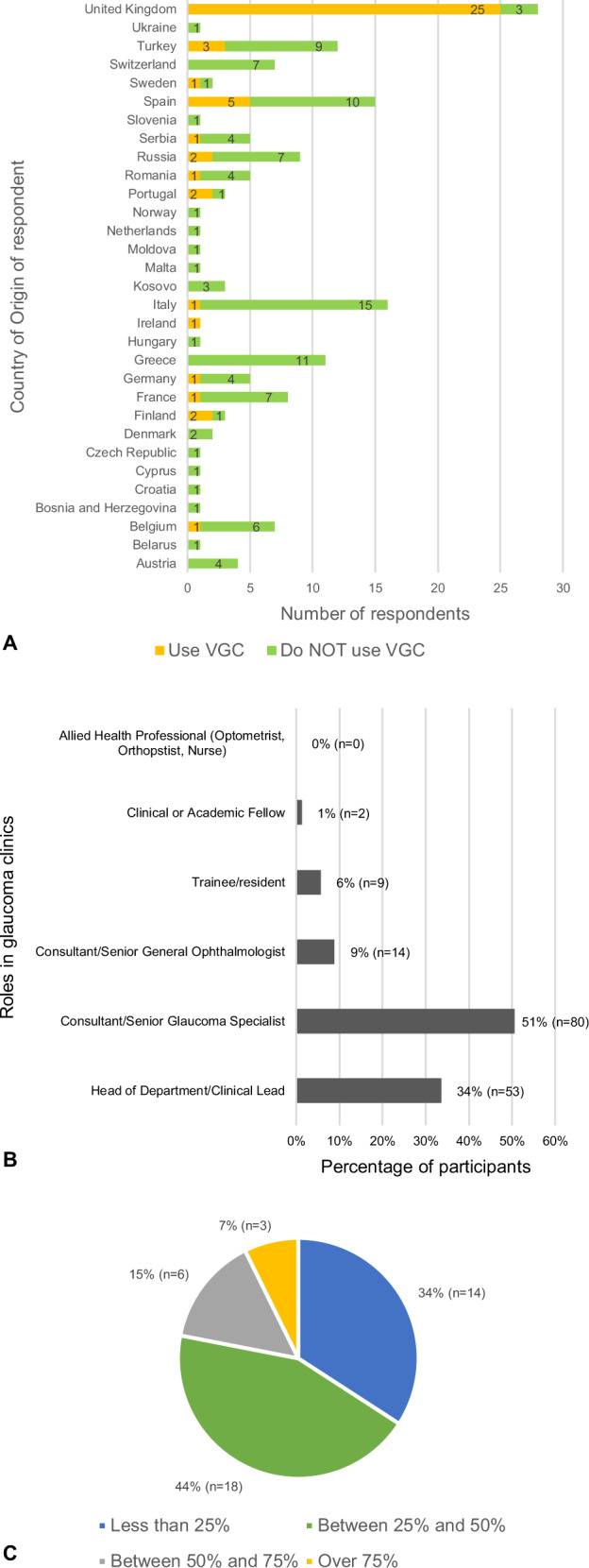
Study demographics. **A** Number of participants per country, stratified by virtual glaucoma clinic use. **B** Main roles of participants in their glaucoma clinics. **C** Proportion of the whole glaucoma population who are followed up at the respondents’ virtual glaucoma clinics.

A total of 169 EGS members answered the survey, reflecting a response rate of 25.4% (664 registered members). 158 (93%) were based in Europe, whilst 11 worked outside Europe and were thus excluded. Of the 158 members, 56% (88) were based in EU-member countries and 44% (70) practiced in non-EU member states. In total, the participants represented 31 European countries (Fig. 1A). The majority of participants (*n* = 93;59%) perform glaucoma clinical activities in teaching or academic hospitals. The rest are based in community hospitals (*n* = 27;17%), district general hospitals (DGHs) (*n* = 11;7%), community clinics (*n* = 18;11%), and private clinics (*n* = 9;6%). Therefore, these responders were typical of the wide-reaching EGS membership, encompassing a range of glaucoma specialists working across different institutions within the geographical boundaries of Europe. The clinical designation of all participants is depicted in Fig. 1B.

47 participants (30%) use VGCs in their glaucoma clinical activities, whilst the remaining 111 (70%) do not. About half of those using VGCs are based in the UK (*n* = 25;53%). A breakdown of participants’ use per country is shown in Fig. 1A. *Of those using VGCs*, 64%(*n* = 30) are based in teaching or academic hospitals, 13%(*n* = 6) in community clinics, 13%(*n* = 6) in DGHs, 6%(*n* = 3) in community hospitals, and 4%(*n* = 2) in private clinics. The difference in VGC use between academic and non-academic institutions was found to be non-significant using the Chi squared test of Independence (*χ*^2^ = 0.6822, df = 1, *p* < 409).

### Use of VGCs

Six of the 47 participants who use VGCs did not divulge further information. Of the remaining 41 respondents, 16(39%) have been using VGCs for over 5 years, 3(7%) for 3–5 years, 15(37%) for 1–3 years, while 7(17%) started using one in the past year. The proportion of patients followed up in VGCs is depicted in Fig. 1C.

**Fig. 2:**
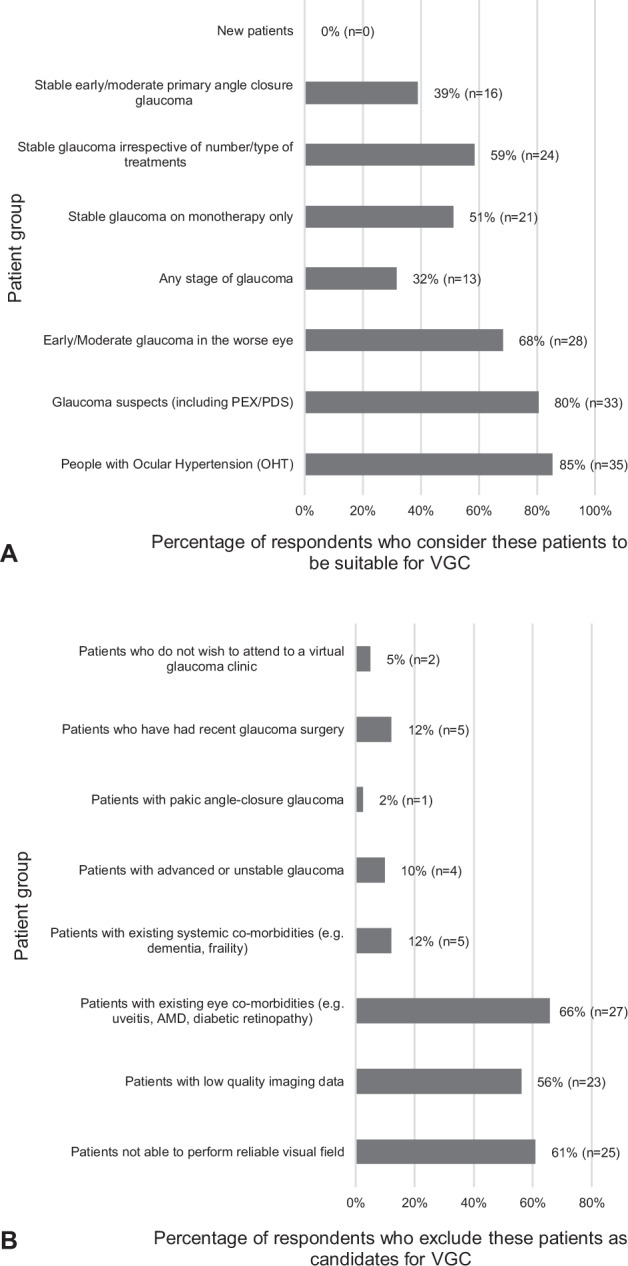
Inclusion and exclusion criteria used by respondents’ virtual glaucoma clinics. **A** Patients considered most suitable for the respondents’ current virtual glaucoma clinics. **B** Patients who are excluded from the respondents’ current virtual glaucoma clinics.

37%(*n* = 15) of respondents stated that patients are mainly referred to VGCs by glaucoma specialists within the hospital service after face-to-face consultation. A further 17%(*n* = 7) stated that most referrals come from hospital-based non-glaucoma specialists, including allied health professionals (AHPs) (optometrists, nurse specialists, orthoptists, etc.). Meanwhile, 12%(*n* = 5) receive referrals directly from the community through general ophthalmologists and 10%(*n* = 4) from community-based AHPs. One respondent stated that *all* follow-ups are reviewed virtually. The remaining 22%(*n* = 9) receive referrals from a mixture of the above. Respondent views on inclusion/exclusion criteria are depicted in Fig. 2.

### Structure of VGC in use

54%(*n* = 22) of VGCs use a remote monitoring model, in which the VGC is located somewhere other than the base hospital, but with physician monitoring of collected data. On the other hand, 20%(*n* = 8) use a parallel monitoring model, where the VGC is hospital-based but non-physician monitoring is performed. The remaining 27%(*n* = 11) use a combination of both.

Nurses (*n* = 20;49%) and ophthalmic technicians (*n* = 19;46%) are the main staff responsible for data collection. Other staff involved include optometrists (*n* = 14;34%), orthoptists (*n* = 6;15%), general non-specialist ophthalmologists (*n* = 5;12%), and health care support workers, e.g. auxiliary nurses (*n* = 1;2%). Interestingly, 27(66%) responders stated that a mixture of professionals is usually involved. With regards to data storage, 68%(*n* = 28) use electronic medical records (EMR), 2.5%(*n* = 1) is paper-based and 22%(*n* = 9) use a hybrid system. Two others (5%) use ‘in-house’ bespoke VGC-specific software and one (2.5%) a dedicated web-based platform.

In 51%(*n* = 21) of VGCs, consultant or lead ophthalmologists are mainly reviewing data and making decisions with this role fulfilled by glaucoma specialists in a further 24%(*n* = 10), and 2%(*n* = 1) each by general ophthalmologists, glaucoma fellows and optometrists with specialist qualifications respectively. A combination of all these professionals make decisions in 17%(*n* = 7) of VGCs.

**Fig. 3:**
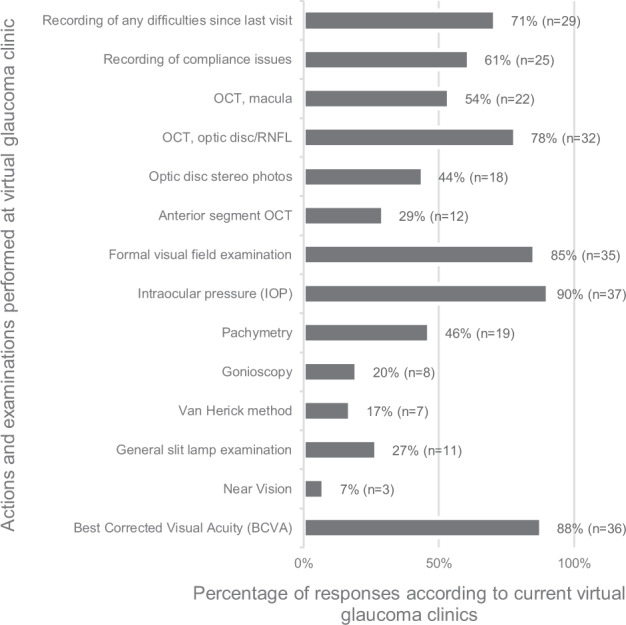
Main parameters. Parameters measured and investigations performed at the respondents’ virtual glaucoma clinics.

Figure 3 shows what actions or investigations are performed at respondents’ VGCs. A total of 33 different test combinations were noted. The commonest combination involved best corrected visual acuity (BCVA) testing, intraocular pressure (IOP) measurement, formal visual field examination, optical coherence tomography (OCT) of optic disc, retinal nerve fibre layer (RNFL) and macula, recording of compliance issues and of any difficulties since last visit.

### Actions taken at the VGC

Patients showing evidence of visual field progression (*n* = 33;80%), increased IOP compared to a predefined target threshold (*n* = 26;63%), or evidence of OCT progression (*n* = 21;51%) are the main groups referred for face-to-face consultation. In 24%(*n* = 10), all patients are eventually booked into a face-to-face review. Patients in whom surgery is a likely treatment option (*n* = 5;12%) and those with medication adherence issues (*n* = 4;10%) are also referred directly for face-to-face review.

### Feedback and professionals’ concerns about VGC use

41% of participants reported VGC-related patient feedback as *very good*, with 44% stating *good* and 15% as *comparable to traditional* care. No participants reported feedback as *poor*. Common clinician concerns include missing comorbidities (54%) and failing to make a diagnosis (32%). Decreased patient adherence (20%) and patients’ complaints for not seeing a doctor (20%) are also prevalent concerns. Others include the slow processing of complex data (5%), detrimental effects on doctor-patient relationships (2%) and the lower priority given to VGCs by the health board management (2%). 15% reported no VGC-related concerns.

### Portion of participants who are not currently using a VGC

88 individuals from the cohort who do not use VGCs further elaborated on their reasons for this. The commonest reasons were lack of experience with virtual clinics (*n* = 41,47%), care models adequately meeting clinical needs (*n* = 37,42%), no appropriate staff for VGCs (*n* = 30,34%) and insufficient time and money to change existing models (*n* = 30,34%). Further reasons included no appropriate clinical space (*n* = 23,26%), lack of investment and resources (*n* = 7,8%), unwillingness to strictly standardise patient examinations (*n* = 7,8%) and software issues (*n* = 1,1%). Only 6%(*n* = 5) stated that they do not believe in the virtual clinic concept, with particular concerns about how IOP measurements and other investigations are obtained virtually and the perceived fact that patients prefer seeing a clinician. When asked whether they are considering implementing VGCs, 29% stated that they are (but not in the near future), whilst 9% said they would be starting one soon. On the other hand, 45% have never considered planning one, 11% have considered it but were unsuccessful and 6% have not found it feasible.

The patient groups considered most suitable for a potential VGC are ocular hypertensives (*n* = 67,80%), glaucoma suspects including pseudoexfoliation and pigment dispersion syndromes (*n* = 47,56%), stable glaucoma on monotherapy (*n* = 47,56%) and early to moderate glaucoma in the worse eye (*n* = 25,30%). Stable early to moderate primary angle closure glaucoma patients are considered to be suitable by 26% (*n* = 22). A lower proportion of respondents also consider stable glaucoma irrespective of treatment or disease severity (*n* = 14,17%) and patients with any stage of glaucoma (*n* = 14,17%) to be suitable for VGCs.

### Effect of SARS-COV2 on virtual, remote or online glaucoma consultations

Professionals using VGCs were asked whether they think the SARS-COV2 pandemic brought about any increase in virtual, remote or online consultations. The majority (*n* = 28;68%) believe this has “definitely increased” with another 6(15%) replying “probably”. On the other hand, 5%(*n* = 2) thought this was “probably not the case” and another 5%(*n* = 2) “definitely not”. Three respondents (7%) were unsure.

From the whole study cohort (126 question responses), the majority (*n* = 69;55%) definitely agreed that the SARS-COV2 pandemic has made virtual clinics worth considering, whilst 39(31%) stated that this was probably true, 6(5%) were unsure, 10(8%) said that this was probably not true, and the remaining 2 participants (2%) stated that this was definitely not the case.

### Main advantages and disadvantages associated with VGC use

**Fig. 4:**
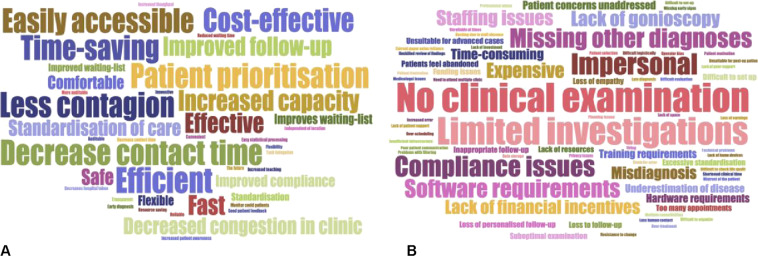
Participants’ main comments about virtual glaucoma clinics, displayed with sizes proportional to the number of times mentioned. **A** Main advantages mentioned. **B** Main disadvantages mentioned.

Finally, the main advantages and disadvantages of VGCs mentioned by all participants are described in Fig. 4.

## Discussion

Monitoring of glaucoma patients is vital for the detection of early progression and initiation of timely treatment to reduce the risk of irreversible visual loss [7]. Unfortunately, due to busy outpatient clinics and hospital-initiated appointment rescheduling, many patients are not being followed up within the recommended window [8]. To complicate matters, the SARS-COV2 pandemic has resulted in thousands of ophthalmology outpatient visit cancellations, hence prolonging the monitoring interval [9, 10]. Viewing ophthalmic data and making clinical decisions remotely has been proposed as the most efficient way of monitoring chronic eye diseases such as glaucoma [11, 12]. In this study, we aimed to assess the uptake of ‘virtual’ patient monitoring systems throughout Europe in the context of glaucoma. To our knowledge, this is the first study to analyse VGC use across different European countries.

Nearly a third of survey participants (30%) are already using VGCs in their day-to-day practice, with 39% of these for more than 5 years. Furthermore, 54% have set-up their VGC within the past 3 years, showing that interest in VGCs is steadily increasing. The commonest VGC model used throughout Europe according to our findings is the asynchronous model, also known as ‘remote monitoring’, similar to previous reports [13]. Another form of asynchronous VGC, where data-gathering occurs within the same hospital setting but non-physicians review it (also known as ‘parallel monitoring’) was not as common amongst our participants. Interestingly, 27% use a mixture of both, indicating that both remote and parallel monitoring may have a role, possibly for different risk categories.

The survey indicates that nurses, ophthalmic technicians and optometrists are the commonest staff who gather data in VGCs throughout Europe, corresponding to previously published VGC models in current use [14–16]. Other respondents mentioned orthoptists, general ophthalmologists and health care support workers, such as auxiliary nurses for this role. Importantly, two-thirds of our VGC cohort utilise a mixture of professionals, promoting a holistic team approach with each individual playing to their own strengths.

Studies have demonstrated that VGCs are safer if a glaucoma specialist reviews the collected data when it comes to diagnosis and risk stratification [17, 18]. In our participants’ VGCs, consultant or lead ophthalmologists are the main clinicians reviewing patient data. However, studies have shown that with experience and training, data interpretation of non-medical staff improves significantly [19, 20], and that management decisions taken by them compare highly to those taken by glaucoma specialists [19–21]. Other studies have shown that clinicians are better equipped at detecting progression if displays of visual field series highlighting information pertaining to progression are analysed [22]. The majority of our participants store data either exclusively in an EMR or a dedicated web-based platform. All these ‘paperless’ or ‘paper-light’ set-ups tend to favour progression analyses using software-based statistical data representation [23]. Only a minority of our participants reported storing data in paper format with a further 22% using a hybrid system.

More than half of our participants’ VGCs receive referrals mainly from their own hospital service, indicating that VGCs are used to supplement this. Other VGCs receive referrals directly from community-based professionals. In these scenarios VGCs could be acting as screening and/or triaging tools, ensuring that the most urgent or complex cases are referred for in-person specialist assessment. For respondents using VGCs, the most suitable patient groups for virtual review are those with ocular hypertension (OHT), glaucoma suspicion, early/moderate glaucoma in the worse eye, stable glaucoma irrespective of number/type of treatments and stable glaucoma on monotherapy only. Concordance between participants was higher the less complex and more stable the disease is, which is similar to published VGC models in current use [15, 18]. On the other hand, participants mostly agreed that new patients should not be seen in VGCs and two-thirds felt that patients with ocular co-morbidities should rather be followed-up traditionally [15, 17]. Interestingly, the effect on the doctor-patient relationship and the slow process when reviewing complex data were not high on our participant’s list. This fits with studies showing similar levels of acceptance and patients’ disease understanding between VGCs and ‘standard’ patients [16]. Most of participants using VGCs reported that patients liked the virtual model.

The investigations done and patient journey through a VGC play a vital role in the effectiveness and safety of a VGC. In our study, participants reported that IOP measurement, BCVA, visual field testing and RNFL and/or optic disc OCT were the commonest investigations undertaken in their VGC.

IOP measurement by Goldmann applanation tonometry (GAT) is regarded by many as the gold standard [24]. However, some authors have found GAT to be potentially inaccurate with others recommending a move towards other IOP measuring techniques [13, 25]. Issues with training and certifying AHPs for GAT may have an impact on the choice of IOP measuring instrument chosen.

Furthermore, participants reported a low usage of methods such as gonioscopy, slit lamp examination, Van Herick and anterior segment OCT (AS-OCT). This is probably due to the fact that they favour VGC use for follow-up. However, when needed the Van Herick method is a relatively quick and easy test to do and learn, whereas AS-OCT could be advantageous for a virtual setting, being non-contact, rapid, and more sensitive at detecting angle closure than gonioscopy [26]. Other studies have identified history-taking and simple advice on common problems such as blepharitis, drop technique and medication side-effects as important steps in a successful VGC [17, 27]. In our participants’ VGCs, recording of new difficulties and compliance issues were also prominent. This wide variation and lack of some tests performed at VGCs indicates that guidelines about VGC structure and recommended investigations are needed. These could be easily tailored to different VGCs to ensure that the setup is tailor-made for the goals of that particular setting, be it screening, follow-up, diagnosis or otherwise.

The efficient functioning of a VGC is dependent on the decision streams made by the reviewer. According to our results, the main patient groups referred for a face-to-face consultation were those showing evidence of visual field progression, increased IOP compared to a predefined target and those with evidence of OCT progression. Furthermore, 24% of participants’ VGC refer all patients for a face-to-face review at some point. This raises the need for a set guideline which stratifies patients into risk progression categories, with specific recommendations accordingly. Such risk-stratification systems can help guide the time frame and type of follow-up appointment that a patient should be given [17, 18]. Although most of these systems are based on clinical evidence of progression, it might be advantageous to adopt a ‘red-flag’ system, also incorporating other patient issues which could affect treatment outcomes, such as medication adherence. In fact, only 10% of our participants’ VGCs refer patients with adherence issues for a face-to-face review. A red-flag system could serve to increase safety and effectiveness of VGCs, whilst leaving reviewers free to tailor recommendations on a case-by-case basis. Attempts at producing VGC guidelines have already been made, such as the work by Kotecha et al. which has produced a set of standards aiming to make VGCs safer [13]. For participants using VGCs, the main concerns are either missing co-morbidities or the actual diagnosis of glaucoma or related conditions. This highlights that patients followed-up virtually should also be seen in-person at certain points, to ensure that important diagnoses are not missed, despite studies indicating that misclassification of events is rare [17]. Interestingly the effect on the doctor-patient relationship and the slow process of reviewing complex data were not high on our participant’s list.

More than two-thirds of survey participants are not currently using VGCs. The main reasons are lack of experience, an adequate system currently in place, no appropriate staff, and insufficient time and money. A recent meta-analysis reported that the mean cost for standard VGC equipment can range from $89,703 to $123,164, excluding additional secure data transfer costs [28]. These start-up expenses, along with maintenance costs, could hinder some healthcare organisations from setting up a VGC. However, on a brighter note using teleglaucoma to screen for glaucoma is even more cost-effective with predicted savings of $27,460 per quality-adjusted life year [29]. Many participants not currently using VGCs have highlighted an interest in using them; only 6% are not interested. Financial re-imbursement could be considered as a means of helping organisations with any inhibitive costs as shown by teleglaucoma projects in Canada and Australia where reimbursements have counterbalanced personnel and technical costs and increased the use of VGCs [30, 31].

Over the past year, the SARS-COV2 pandemic has brought about significant changes in the way we practice medicine. Worldwide, a rapid increase in virtual consultations in order to minimise contact and continue care provision has been reported [6, 9]. The majority of survey participants using VGCs stated that they believe virtual consultations have increased during the pandemic. Furthermore, the majority think that the pandemic has made the need for virtual consultations even more important. This highlights the potentially increased role of virtual clinics for glaucoma management in the coming years.

Our study has a number of limitations. A survey-style can introduce sampling errors about country-specific VGC-use. Some countries indeed had significantly lower response proportions. Furthermore, a selection bias could be present as respondents may have been more inclined towards favouring VGCs. The majority of participants perform their glaucoma clinical activities in teaching hospitals, with nearly two-thirds of individuals using VGCs working in academic institutions. However, two-thirds of all participants from academic institutions do not use VGCs and no significant difference was found in VGC-use between academic and non-academic-based participants (*p* = 0.409). Finally, a direct cost-benefit analysis was difficult to perform and no direct inferences could be made about the cost-benefit of VGCs in current use.

In summary, this survey shows that a significant proportion of glaucoma specialists, are already using VGCs throughout Europe. The commonest VGC model used is the asynchronous model, with the vast majority moving away from paper-based systems. Allied health professionals are the commonest data-gathering staff whilst consultant or lead ophthalmologists are the main reviewers of clinical data. IOP measurement, BCVA, visual field testing and RNFL/optic disc OCT are the commonest investigations performed, whilst gonioscopy, slit lamp examination and AS-OCT are not used as much. A quarter of our participants refer everyone for a face-to-face review at some point, but the majority will only refer patients showing evidence of visual field or OCT progression or those who have increased IOP compared to a predefined target. For those not using VGCs, the main reasons were lack of experience, adequate current systems, no appropriate staff and insufficient time or money. Potentially, financial re-imbursement, consensus guidelines and more awareness might aid further VGC uptake in the future.

## Summary

### What was known before

- Glaucoma related burden is increasing with resulting longer patient waiting times, made worse since the advent of the SARS-CoV-2 pandemic.
- Teleophthalmology and virtual clinics have been shown to increase hospital efficiency and productivity, allowing better utilisation of diagnostic and clinical resources according to severity and need.
- The most commonly described virtual glaucoma clinic (VGC) model is asynchronous, in which ophthalmic staff gather glaucoma-relevant data in an outpatient setting, with subsequent separate clinician review. However, most studies have focused on VGC set-ups based solely in the UK, and prior to the COVID pandemic.

### What this study adds

- This is currently the largest published clinician review of VGCs and also the first study to analyse their use across different European countries.
- The study outlines the differences in utilisation of VGCs across Europe, including time since adoption, changing trends, and reasons behind their implementation (or lack thereof).
- The study also delineates the characteristics of various set-ups in various countries and explores clinician perceptions across the continent on VGC format, benefits, target patient groups and the effect of the SARS-CoV-2 pandemic.

## Supplementary information

Supplementary Table

Supplementary Table Caption

## Data Availability

All data generated or analysed during this study are included in this published article and its supplementary information files.
