# Supplementary material for: The current use of glaucoma virtual clinics in Europe
Source: Eye (Lond). 2022 Jun 11;37(7):1350–6. doi: 10.1038/s41433-022-02111-5 (PMC9188015; doi:10.1038/s41433-022-02111-5)
Supplement: Supplementary file 1 — Supplementary Table [file 41433_2022_2111_MOESM1_ESM.docx]

| **Combination** | **Number of participants** |
| --- | --- |
| BCVA, Intraocular pressure, Formal visual field examination, OCT-optic disc/RNFL, OCT-macula, Recording of compliance issues, Recording of any difficulties since last visit | **3** |
| BCVA, Intraocular pressure, Formal visual field examination, OCT-optic disc/RNFL, Recording of compliance issues, Recording of any difficulties since last visit | **2** |
| BCVA, Pachymetry, Intraocular pressure, Formal visual field examination, Anterior segment OCT, OCT-optic disc/RNFL, Optic disc stereo photos, OCT-macula, Recording of compliance issues, Recording of any difficulties since last visit | **2** |
| BCVA, Pachymetry, Intraocular pressure, Formal visual field examination, Anterior segment OCT, OCT-optic disc/RNFL, OCT-macula, Recording of compliance issues, Recording of any difficulties since last visit | **2** |
| BCVA, Pachymetry, Intraocular pressure, Formal visual field examination, Anterior segment OCT, OCT-optic disc/RNFL, OCT-macula, Recording of any difficulties since last visit | **2** |
