# Supplementary material for: The current use of glaucoma virtual clinics in Europe
Source: Eye (Lond). 2022 Jun 11;37(7):1350–6. doi: 10.1038/s41433-022-02111-5 (PMC9188015; doi:10.1038/s41433-022-02111-5)
Supplement: Supplementary file 2 — Supplementary Table Caption [file 41433_2022_2111_MOESM2_ESM.docx]

**Table 1:** Main combinations of actions performed at VGC noted from the responses.
